# Targeting HIV-1 Protease Autoprocessing for High-throughput Drug Discovery and Drug Resistance Assessment

**DOI:** 10.1038/s41598-018-36730-4

**Published:** 2019-01-22

**Authors:** Liangqun Huang, Linfeng Li, ChihFeng Tien, Daniel V. LaBarbera, Chaoping Chen

**Affiliations:** 1 Department of Biochemistry and Molecular Biology, Colorado State University, Fort Collins, Colorado USA; 2 Department of Pharmaceutical Sciences, Skaggs School of Pharmacy and Pharmaceutical Sciences, University of Colorado Anschutz Medical Campus, Aurora, Colorado USA

## Abstract

HIV-1 protease autoprocessing liberates the free mature protease from its Gag-Pol polyprotein precursor through a series of highly regulated autoproteolysis reactions. Herein, we report the development and validation (Z’ ≥ 0.50) of a cell-based functional assay for high-throughput screening (HTS) of autoprocessing inhibitors using fusion precursors in combination with AlphaLISA (amplified luminescent proximity homogeneous assay ELISA). Through pilot screening of a collection of 130 known protease inhibitors, the AlphaLISA assay confirmed all 11 HIV protease inhibitors in the library capable of suppressing precursor autoprocessing at low micromolar concentrations. Meanwhile, other protease inhibitors had no impact on precursor autoprocessing. We next conducted HTS of ~23,000 compounds but found no positive hits. Such high selectivity is advantageous for large-scale HTS campaigns and as anticipated based on assay design because a positive hit needs simultaneously to be nontoxic, cell permeable, and inhibiting precursor autoprocessing. Furthermore, AlphaLISA quantification of fusion precursors carrying mutations known to cause resistance to HIV protease inhibitors faithfully recapitulated the reported resistance, suggesting that precursor autoprocessing is a critical step contributing to drug resistance. Taken together, this reported AlphaLISA platform will provide a useful tool for drug discovery targeting HIV-1 protease autoprocessing and for quantification of PI resistance.

## Introduction

HIV-1 protease (PR) is one of the three viral encoded enzymes essential for productive viral replication. In the infected cell, the unspliced genomic RNA functions as the mRNA to mediate translation of the Gag and Gag-Pol polyprotein precursors with the ratio between the two controlled by a regulated ribosomal frameshift occurring at the end of the nucleocapsid coding sequence^1–3^. Within the Gag-Pol polyprotein, the PR is embedded between an upstream peptide and the downstream reverse transcriptase (RT)^3^. The upstream peptide is called the transframe region (TFR) or p6*^4,5^ and its coding sequence overlaps with the p6 in the Gag reading frame. The Gag and Gag-Pol polyproteins co-assemble into viral particles, which bud off from the infected cell^6–8^. Upon or shortly after virion release, the Gag-Pol polyprotein is triggered to undergo autoproteolysis resulting in the liberation of the free mature PR; a process generally referred to as PR autoprocessing. There are at least 10 cleavage sites in Gag and Gag-Pol polyproteins that can be processed by the mature PR at various rates and modulations^3,4,9–14^. Concerted proteolysis of these sites is required for proper virion maturation that in turn determines viral infectivity^10,15–24^.

From the Gag-Pol precursor to the free mature protease, HIV-1 protease autoprocessing is a complicated process in which the Gag-Pol precursor must function as both the catalyst and substrate before any mature PR becomes available. Extensive research efforts have focused on structural and enzymatic characterization of the mature PR, which has led to successful development of ten FDA-approved PIs. All PIs share the same mechanism of action and bind to the catalytic site of the mature PR with high affinities^25–27^. However, the protease autoprocessing mechanism remains largely undefined. There are at least two autoproteolysis reactions essential to liberate mature PR: one at the N-terminus between p6* and PR, and the other at the C-terminus between PR and RT. Mutagenesis analyses demonstrated that the PR-RT fusion results from blocking the C-terminal cleavage site, which maintains the enzymatic activities vital for productive viral replication. This suggests that C-terminal extensions have less impact on viral infectivity^28^. Conversely, blocking N-terminal cleavage leads to detection of a p6*-PR fragment in viral particles that have been shown to be non-infectious^21^. It is interesting to note that several other Gag and Gag-Pol cleavage sites were also processed in these viral particles, demonstrating proteolysis activities by the p6*-PR fragment or other precursors in the absence of mature PR. Meanwhile, the p6*-PR is clearly insufficient at producing infectious viral particles as mature PR is required for complete Gag processing. Additionally, p6* removal from a recombinant p6*-PR protein coincides with the appearance of mature PR activity^25,29^. Collectively, results of these studies have established p6*-PR as a miniprecursor that is enzymatically different from the mature PR^3,21,29–35^.

We have established a cell-based assay to study the autoprocessing mechanism by expressing fusion precursors in transfected mammalian cells^3,32–34^. A typical fusion precursor consists of the p6*-PR miniprecursor (derived from the NL4-3 strain) sandwiched between GST and a small epitope peptide such as Flag. This assay allows examination of precursor autoprocessing reactions inside of mammalian cells, which is different from the *in vitro* assay using the recombinant p6*-PR purified out of *E. coli* inclusion body followed by protein re-folding^29,36,37^. With our assay, we have demonstrated that the currently available HIV- 1 protease inhibitors (PIs) are much less effective at suppressing precursor-mediated autoprocessing than inhibiting mature protease activity^33,34,38^, which is consistent with other reports^29–31,39^, confirming that the precursor is enzymatically different from the mature PR. Additionally, we reported that precursor autoprocessing is a context-dependent process such that different fusion precursors carrying different tags and/or mutations outside the PR coding region released autoprocessing products with distinct enzymatic properties^34,38^.

To enable identification of novel inhibitors that selectively target the precursor-mediated autoproteolysis, we employed amplified luminescent proximity homogeneous assay ELISA (AlphaLISA) for quantification of autoprocessing efficiency using crude cell lysates to convert the cell-based assay into a 384-well plate format for high throughput screening (HTS) drug discovery. This study compared quantification results from conventional western blotting and AlphaLISA analyses. We began by evaluating known HIV PIs for their efficacies at suppressing precursor autoprocessing and determined performance parameters such as Z’-factor, and S/N ratios for assay validation^40,41^. We then carried out HTS of ~23,000 diverse small molecule compounds. Additionally, we examined autoprocessing of fusion precursors carrying mutations identified from patients experiencing PI resistance to determine the role of precursor autoprocessing in drug resistance development. Results of these analyses collectively validated this newly developed AlphaLISA platform as a useful tool for HTS drug discovery targeting precursor autoproteolysis and to quantify PI resistance.

## Results and Discussion

### Quantification of precursor autoprocessing by amplified luminescent proximity homogeneous assay ELISA (AlphaLISA)

**Figure 1:**
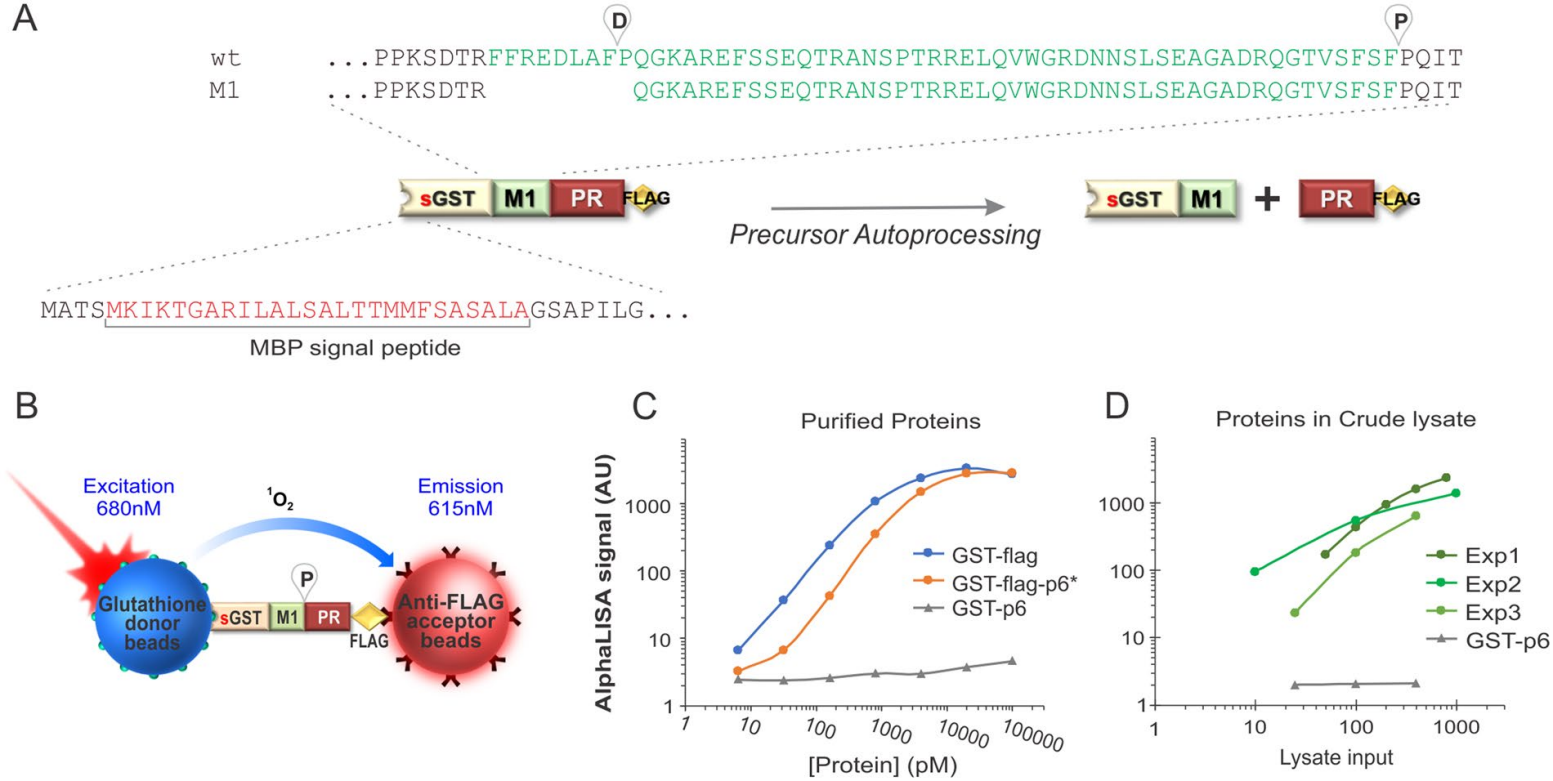
AlphaLISA detection. (**A**) Schematic illustration of the fusion precursors used for autoprocessing study in this report. (**B**) Detection principle of the full-length fusion precursor by AlphaLISA. (**C**) AlphaLISA detection of the indicated purified proteins spiked into the crude lysates made from the mock transfected cells at serial dilutions. (**D**) AlphaLISA detection of the target proteins in crude lysates. HEK 293T cells were transfected with the expression plasmids (GST-Flag or GST-p6), lysed *in situ* with the AlphaLISA assay buffer, and further diluted at serial dilutions prior to AlphaLISA detection. The lysate input was calculated based on cell confluency and buffer volume at the time of lysis to represent number of cells used for AlphaLISA detection.

We previously established a cell-based assay to study the precursor autoprocessing mechanism inside mammalian cells by expressing fusion precursors with the p6*-PR miniprecursor sandwiched between various fusion tags^32–34,38,42,43^. Domain organization of a typical fusion precursor is illustrated in Fig. 1A. The NL4-3 derived p6*-PR has two autoproteolysis sites. One is between p6* and PR, designated as the proximal (P) site, which is equivalent to the N-terminal processing site essential for liberation of mature PR. The other one is located at the N-terminal region of p6*, defined as the distal (D) site. In the context of GST fusion, precursor autoprocessing at these two sites appears to be independent of each other^32,33^ and we thus engineered M1-PR precursor to specifically focus on the proximal site autoprocessing. Also, we recently reported that precursor autoprocessing is context-dependent and the maltose binding protein signal peptide at the N-terminus leads to autoprocessing outcomes similar to those observed with viral particles^34^. Therefore, we engineered our fusion precursors all carrying the signal peptide in this study (Fig. 1A).

Precursor autoprocessing efficiency is reversely correlated with the amount of the full-length (FL) fusion precursor, *i.e*., inhibiting autoprocessing leads to accumulation of the FL precursors inside cells. We thus chose AlphaLISA, a label-free and wash-free detection platform^44–47^ for autoprocessing quantification. The key components of AlphaLISA are latex-based donor and acceptor beads that are small enough to remain suspended in solution and can be conjugated with diverse molecules and antibodies to mediate specific target binding. In the crude cell lysates, the FL fusion precursor would mediate complex formation consisting of glutathione-coated donor beads at the N-terminus and anti-FLAG coated acceptor beads at the C-terminus (Fig. 1B). The donor beads contain a photosensitizer, phthalocyanine, which upon excitation at 680 nm converts ambient oxygen to singlet oxygen that is short-lived (t_1/2_ = 4 µ sec) due to its high reactivity and thus only diffuses ~200 nm in solution. On the other hand, AlphaLISA acceptor beads contain europium, which emits chemiluminescence peaked at 615 nm upon reaction with singlet oxygen. Consequently, the chemiluminescent signal is proportional to the amount of FL precursor under optimized conditions. Effective autoprocessing cleaves the precursor such that the average distance between a donor and an acceptor bead is greater than 200 nm; no chemiluminescence produced. AlphaLISA sensitivity is cooperatively determined by the binding affinities between the target analyte and the antibodies, or molecules immobilized onto the beads, as well as solution conditions.

We first examined AlphaLISA detection sensitivity using purified proteins at serial dilutions spiked into the lysates made from mock transfected cells (Fig. 1C). AlphaLISA signal was linearly correlated to GST-Flag concentration over about three orders of magnitude with a detection limit as low as 30 pM. Another purified protein, GST-Flag-p6* (with the Flag tag placed in the middle), showed a slight right shift but still displayed concentration-dependent detection. Importantly, a GST-p6 control protein lacking a Flag tag generated only background levels of signal, confirming high specificity and sensitivity of AlphaLISA detection. Crude cell lysates made from three independent transfections expressing GST-Flag also displayed a linear correlation between AlphaLISA signal and the lysate input (Fig. 1D). It is worth noting that AlphaLISA signal plateaued as the target protein input reached to certain amounts. This is mainly due to the “hook effect”, a phenomenon common to detections involving saturable regents such as donor and acceptor beads. When the target proteins are less than the beads available in the mixture, AlphaLISA signal is linearly correlated with the amount of target protein. When the target proteins are more than the beads, AlphaLISA signal no longer increases and even decreases as an excess of the target oversaturates the limiting beads preventing complex formation. Nonetheless, these pilot experiments demonstrated sensitive AlphaLISA detection using crude cell lysates.

We then considered and experimentally determined two assay parameters for optimal and effective AlphaLISA quantification. According to AlphaLISA principle, the more beads we use, the wider detection window we have (before the hook point). However, using more beads are cost ineffective as these beads are rather expensive. Therefore, we titrated the minimal amount of each bead (donor and acceptor) that provided big enough detection window (>50-fold changes) using purified GST-Flag-p6* as the target. This control was also included in each experiment for system validation hereinafter. Another consideration was the amount of target protein used for the assay, which was collectively determined by the number of transfected cell and transfection efficiency. Because of the hook effect, it would be critical to avoid using too many target proteins for the assay. We examined AlphaLISA signal in correlation with plating density and determined that bulk transfected cells seeded ~25% confluency in 384-well plates produced adequate amounts of FL precursors (upon PI suppression) for sensitive AlphaLISA detection (S/N > 15). As shown in Sup. Fig. S1, seeding more cells (at 50% confluency) displayed less separation between the positive and negative samples (S/N < 10) although both showed similar response curves. Subsequently, we standardized the assay by seeding bulk transfected cells at ~25% confluency.

**Figure 2:**
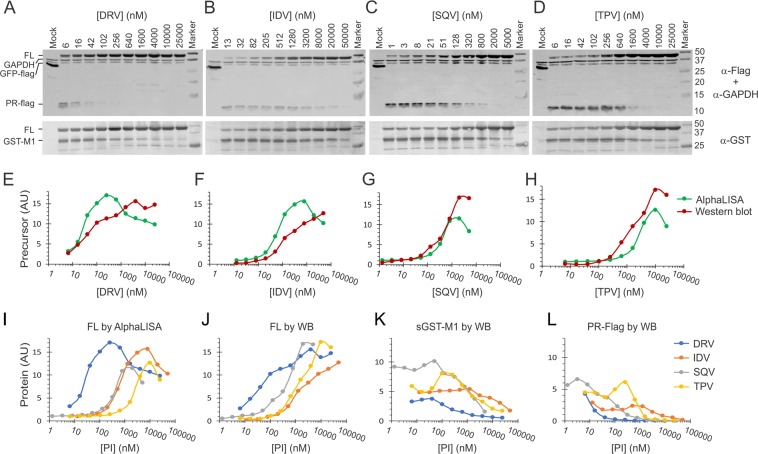
Quantification of precursor autoprocessing. (**A**–**D**) Detection of precursor autoprocessing by western blotting. The transfected cells were treated with the indicated PIs and the post-nuclear cell lysates were analyzed by SDS-PAGE and western blotting. The images were from four gels analyzed in parallel at the same time. The upper panel was visualized with mouse anti-Flag and anti-GAPDH followed by IR800 anti-mouse probing; the lower panel was visualized with rabbit anti-GST followed by IR700 anti-rabbit probing. Duel detection of IR700 and IR800 channels with a LI-COR scanning unit allowed simultaneous examination of multiple bands in the same gel. Band intensity normalized to GAPDH signal of the same lane was determined to represent protein amount for quantitative analysis. The full images of these blots (Sup. Fig. S2) can also be found in the Supplementary Information file. (**E**–**H**) Comparison of precursor quantification by western blotting (red lines) and AlphaLISA (green lines) in respond to various PIs. (**I**–**L**) PI effects on the FL fusion precursor (**I** and **J**), sGST-M1 (**K**) and PR-Flag (**L**) autoprocessing products, quantified by AlphaLISA or western blotting.

We also compared AlphaLISA and western blotting detection by examining precursor autoprocessing in response to HIV PI treatment (Fig. 2). Western blotting allowed detection of the FL fusion precursor as well as autoprocessing products (sGST-M1 and PR-Flag) but had a low throughput power (Fig. 2A–D). Consistent with a previous report, the mature PRs released from sGST fusions were readily detectable in the absence of any PI^34^. When treated with increasing PI concentrations, accumulation of the FL precursor became evident as the autoprocessing reaction was suppressed, confirming the inverse correlation between FL precursor detection and autoprocessing efficiency. AlphaLISA detection displayed a bell-shaped profile as a function of PI concentration (Fig. 2E–H, green lines). The left half of the response curve reflected accumulation of the FL precursor resulted from autoprocessing suppression by HIV PIs before the hook point, thus was used for IC_50_ determination. After the hook point, signal decline was observed to various extends. Darunavir treatment showed a slight dip in AlphaLISA signal and then plateaued; whereas others exhibited a decline towards the end of test window. In addition to the hook effect, we suspected that diverse PI cytotoxicity (especially at high micromolar concentrations) could be another contributor to the observed differences. Nevertheless, the four tested PIs exhibited various potency at suppressing precursor autoprocessing with their apparent IC_50_s ranging from ~25 to 3000 nM (Fig. 2I). Darunavir demonstrated the most potent inhibition; indinavir and saquinavir were similar showing submicromolar IC_50_s; tipranavir was the weakest at suppressing precursor autoprocessing. Our data is consistent with other report showing that various PIs exhibit diverse efficacies at inhibition precursor autoprocessing with low micromolar IC_50_s^31,39^.

A side-by-side comparison of AlphaLISA to western blotting quantification demonstrated consistent results in detection of the FL precursor (Fig. 2E–H). We noted that AlphaLISA curves were left shifted in the cases of darunavir and indinavir. Subsequently, the IC_50_ values identified by AlphaLISA were slightly lower than those determined by western blotting. We attributed this discrepancy to the difference in detection principle between these two methods because AlphaLISA has a built-in amplification step when chemiluminescence signal is produced by singlet oxygen making it significantly more sensitive in FL precursor detection. In the cases of saquinavir and tipranavir, AlphaLISA peak heights were lower than darunavir and indinavir; whereas western blotting showed similar levels of FL precursor accumulation. We postulated that other tag-containing fragments (*i.e*., autoprocessing products such as sGST-M1 and PR-Flag) in the lysates competed for bead binding and hence reduced peak height. Indeed, we detected more sGST-M1 and PR-Flag products in lysates from saquinavir or tipranavir treated cells than from darunavir and indinavir treated cells (Fig. 2K,L). Taken together, both methods were capable of quantitative evaluation of autoprocessing efficiency although the absolute IC_50_ values may vary slightly from one method to another.

### AlphaLISA Optimization

We further evaluated other assay parameters to establish an effective HTS platform. Transfected cells expressing the target precursor were treated with 5 µM indinavir in the presence of DMSO up to 2% final concentration were examined to determine its cytotoxic effect. A decline in target precursor detection by western blotting was linearly correlated with DMSO concentration to 1.25% final concentration, after which a drastic drop was observed. Also, cytotoxicity (cell floating or detaching) became obvious when DMSO concentration was >0.5% although the FL precursor remained detectable. Therefore, we kept the DMSO final concentration <0.5% whenever possible. Another assay optimization was to include a trace amount of eGFP encoding plasmid for co-transfection and measured fluorescence intensity of each plate to represent cell numbers in each well. Importantly, fluorescence detection does not interfere with AlphaLISA signal detection as their excitation and emission spectra do not overlap. This allowed for multiplex determination of both cell density and the FL precursor in each well to facilitate identification and exclusion of compounds that present a low AlphaLISA signal due to cytotoxicity. We experimentally determined a linear correlation between GFP signal and cell density when the eGFP encoding plasmid was mixed with the precursor encoding plasmid at a 3:97 ratio by weight. Collectively, we optimized a protocol for autoprocessing quantification in 384-well format.

### HTS of several compound libraries

**Figure 3:**
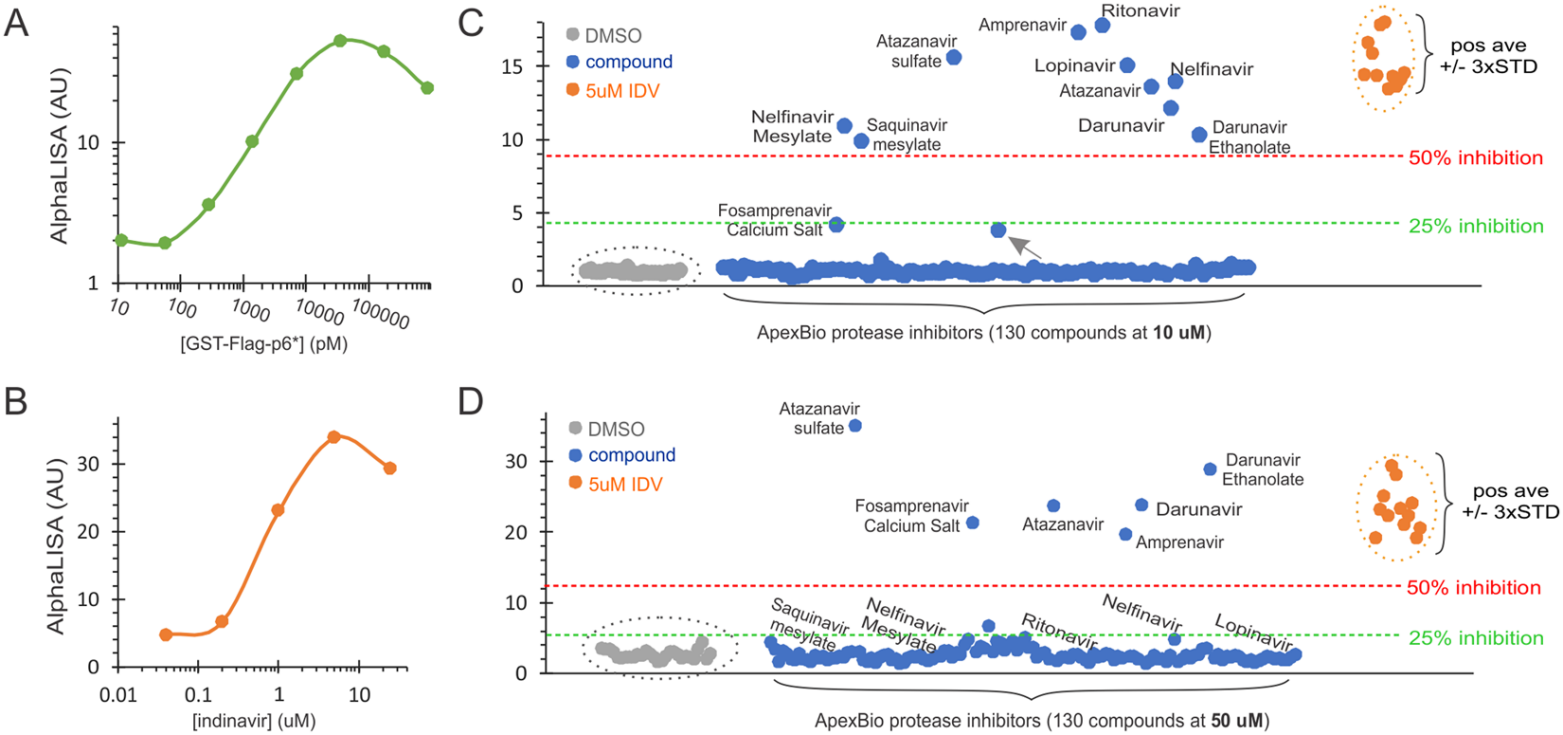
AlphaLISA screening of ApexBio protease inhibitor library. The bulk transfected cells seeded in 384-well plates were treated with the indicated compounds for about 24 hrs before AlphaLISA analysis. (**A**) Purified GST-Flag-p6* protein at a serial dilution spiked into the lysates made from DMSO treated cells was measured as a system control. (**B**) AlphaLISA quantification of transfected cells treated with increasing indinavir. (**C** and **D**) AlphaLISA screening at 10 µM with 0.1% DMSO (upper) or 50 µM with 0.5% DMSO (lower) in duplicates. Each dot was the average of each sample divided by the average of DMSO alone controls (grey dots) to reflect the amounts of the fusion precursor in the crude lysates. Cells treated with 5 µM indinavir (orange dots) were tested in parallel as positive controls.

**Figure 4:**
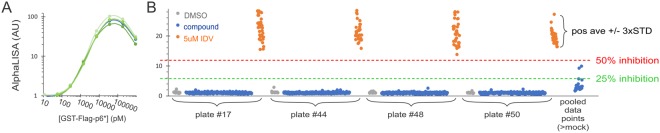
Representative screen data of a 20 K diversity set. The bulk transfected cells seeded in 384-well plates were treated with the compound at a single dose (50 µM in the presence of 0.5% DMSO) for about 24 hrs before AlphaLISA analysis. (**A**) Signals of purified GST-flag-p6* at a serial dilution spiked into the DMSO treated lysates from four independent experiments were plotted together to illustrate system reproducibility. (**B**) Data from four screen plates plus those from other plates showing higher than DMSO controls were pooled together to the far-right end. Each dot represents a sample with grey dots being DMSO controls and orange dots being 5 µM indinavir controls.

We subsequently screened a few compound libraries to evaluate feasibility and performance of the AlphaLISA platform for discovery of autoprocessing inhibitors. Purified GST-Flag-p6* spiked into the lysate made from transfected cells treated with DMSO was included in each HTS experiment as a system control to ensure sensitive detection (Figs 3A and 4A). Because the DMSO treated lysates contained autoprocessing products (GST-M1 and/or PR-Flag) that competed with the spiked protein for bead binding, the resulting detection was ~10-fold less sensitive than that obtained using lysates made from mock transfected cells (Fig. 1B). Otherwise, these controls showed consistent linear correlation between AlphaLISA signal and spiked protein with a satisfactory detection window before reaching to the hook point (Figs 3A and 4A).

ApexBio Protease Inhibitor Library is a collection of 130 protease inhibitors that target a variety of proteases including dipeptidyl peptidase 4 (DPP-4), proteasome, caspases, gamma secretase, etc. There are 11 known HIV-1 PIs in the library. These compounds were first screened at 10 µM final concentration with 0.1% DMSO at duplicates. Transfected cells treated with 0.1% DMSO were included as negative controls and those with 5 µM indinavir in the presence of 0.1% DMSO as positive controls. Cells treated with increasing concentrations of indinavir were also tested in parallel to determine the dose response profile (Fig. 3B). Once again, AlphaLISA signal was linearly correlated with indinavir concentration from 0.2 to 5 µM and then decreased at 25 µM treatment. Both hook effect and cytotoxicity of indinavir at high micromolar concentrations could be the causes as cell detachment became obvious at this concentration. Out of 35 DMSO treated and 30 indinavir (5 µM) treated samples, we calculated the average values of the positive and negative controls, and standard deviations of both, to be 19.1 and 1.0, 2.7 and 0.1, respectively, which gave a Z’ factor of 0.52, indicating an excellent assay for HTS^41^. All HIV PIs in the library but one inhibited autoprocessing more than 50% above the red threshold (Fig. 3C); other protease inhibitors had no influence on precursor autoprocessing. Fosamprenavir calcium salt, a pro-drug of amprenavir, suppressed precursor autoprocessing at ~25%. This result was as expected because PIs are less effective at suppressing precursor autoprocessing than inhibiting mature PR and thus require micromolar concentrations to inhibit precursor autoprocessing^22,30,33,39^. One compound showed a high and a low AlphaLISA signal, resulting in an average that was higher than the negative control average. A retest of this compound confirmed it to be negative. We also observed similar false positive data in DMSO-treated controls at <0.1% rates. We therefore suggested that these false positive noises are rather sparse and can be easily ruled out by a retesting.

The same library was screened again at 50 µM with 0.5% DMSO in duplicate (Fig. 3D) to determine if other protease inhibitors with low potency could partially inhibit autoprocessing at high concentrations. The protease inhibitors in the library, excluding known HIV PIs, showed no impact on precursor autoprocessing. Compared to the 10 µM screen, five HIV PIs (amprenavir, atazanavir, atazanavir sulfate, darunavir, and darunavir ethanolate) showed consistent suppression of precursor autoprocessing, confirming their relatively potent efficacy and low cytotoxicity (at ≤50 µM). Five other PIs (lopinavir, nelfinavir, nelfinavir mesylate, ritonavir, and saquinavir mesylate) displayed AlphaLISA signals at 50 µM lower than at 10 µM, approaching to the negative controls. We speculated cytotoxicity of these PIs at higher concentrations to be a plausible reason. Indeed, we observed significant cytotoxicity (cell detachment and floating) in transfected HEK 293T cells treated with saquinavir at >10 µM, which is consistent with reports demonstrating similar observations^48–50^. Both darunavir ethanolate and fosamprenavir calcium salt showed AlphaLISA signals at 50 µM higher than at 10 µM, suggesting that these two compounds need higher concentrations to achieve effective suppression. This pilot screening confirmed that HIV-1 PIs can inhibit precursor autoprocessing at micromolar potency while other protease inhibitors do not.

We next screened NIH clinical collection I (450 compounds), collection II (281 compounds), a spectrum collection of 2320 compounds containing 50% approved drugs, 30% diverse natural products, and 10% bioactive compounds. We chose to screen them at 12.5 µM because precursor autoprocessing is difficult to suppress and some known HIV PIs only partially inhibit autoprocessing even at 10 µM (Fig. 3C). Unfortunately, these compounds were provided to us at 1 mM in 100% DMSO, resulting in 1.25% final DMSO concentration, which was higher than the desirable <0.5% concentrations. We experimentally evaluated the impact of 1.25% DMSO and found that it introduced wider variations to all the samples, but nonetheless the positive controls stayed positive in our assay (Sup. Fig. S3). This suggested that the assay can pick up positive hits even under suboptimal conditions. Indeed, screening of these three libraries at 12.5 µM with 1.25% DMSO identified indinavir and ritonavir, two known HIV PIs, and other three promiscuous false positives (methylene blue, protoporphyrin IX, and hematoporphyrin) that frequently showed up as hits in many screens deposited to the PubChem database. Taken together, these screens revealed satisfactory performance of the AlphaLISA platform. Under optimized conditions, we observed signal to noise (S/N) ratios > 15, CV < 15%, and Z’ factors between 0.5 and 0.7, which inferred a separation of >10 times standard deviations between the positive and negative means.

With the optimized and validated HTS assay in hand, we performed a screen of a 20,000-compound diversity set from Life Chemicals (LC), which contain various drug-like chemical scaffolds. The LC library was screened at 50 µM in a single dose in the presence of 0.5% DMSO. Figure 4B shows data from four representative plates along with the positive and negative controls, including data points from other screened plates that displayed higher than negative control signal. By setting the thresholds at 50% (red dash line) or 25% (green dash line) of the corresponding positive average, we found no hit above 50%, and a few between the 25% and 50% cutoffs. We cherry-picked the compounds showing 20–50% inhibition from the library, retested them with the same primary assay at serial dilutions, and found no confirmed hits as they were marginally positive to start with. This HTS exhibited satisfactory performance with an impressive selectivity. To the best of our knowledge, this is the first functional assay amenable for HTS, which would facilitate the discovery of novel autoprocessing inhibitors.

### Sensitive quantification of PI resistance at the precursor autoprocessing step

**Figure 5:**
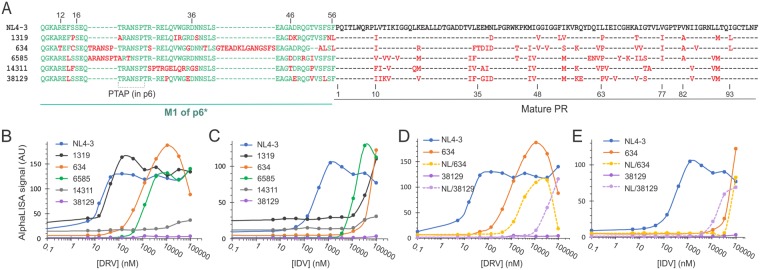
AlphaLISA quantification of PI resistance. (**A**) The M1-PR sequences of the tested constructs with the corresponding patient ID^51^. The green hyphens in the p6* region denote gaps as the length varies; the black hyphens in the mature PR region denote the same amino acids as in NL4-3. Mutations differing from the NL4-3 sequence are highlighted in red. HEK 293T cells transfected with the indicated precursor expression plasmids were seeded in 384-well plates and treated with darunavir (**B** and **D**) or indinavir (**C** and **E**) at increasing concentrations up to 100 µM at duplicates. The AlphaLISA signal of each well was measured after ~24 hrs of incubation and the average values (arbitrary unit) were plotted as a function of PI concentration. The hybrids carrying the M1 region from NL4-3 and the mature PR from PI resistant mutants were labeled as NL/patient ID. The graphs are representative of three independent experiments.

The emergence of drug resistance caused by occurrence and accumulation of HIV-1 mutations in patients under cART (combinational antiretroviral therapy) is an ongoing concern that diminishes treatment efficacy. Extensive sequencing analysis has identified diverse mutations associated with drug resistance, but the underlying mechanism remains largely elusive. The PhenoSense® HIV drug resistance assay is a well-accepted approach for quantification of PI resistance. This assay involves construction of a test vector carrying mutations identified from patients experiencing PI resistance, production, and testing of the resulting viruses in comparison to a reference virus (*e.g*., NL4-3). A ratio of the drug concentration needed to inhibit the test virus relative to that of the reference virus, *i.e*., fold change in IC_50_, is reported to denote PI resistance; higher values correlate to stronger resistance (Table 1). It is worth noting that this reported drug resistance collectively reflects mutation effects on multiple processes such as virus production, Gag processing efficiency, replication competency, and infectivity etc. In contrast, the AlphaLISA specifically measures the precursor-mediated autoprocessing reaction. We sought to see if our AlphaLISA platform could be used to quantify mutation effects on precursor autoprocessing and thus examined fusion precursors carrying mutations identified in patients experiencing PI resistance^51^. Of the 14 reported prototypical infectious molecular clones, we selected five with each containing multiple and diverse mutations in both p6* and PR sequences (Fig. 5A). The corresponding M1-PR coding region was subcloned into the sGST-M1-PR-flag backbone and the resulting constructs were compared to the NL4-3 control in the presence of different HIV PIs.

**Table 1:** Quantification of PI resistance.

Group and patient ID	darunavir		indinavir	
	^a^Fold change by PhenoSense®	^b^Fold change by AlphaLISA	^a^Fold change by PhenoSense®	^b^Fold change by AlphaLISA
1319	0.4	1.7	38	≥100
634	39	43	≥200	≥200
6585	112	52	88	~80
14311	>200	>1000	48	>700
38129	>200	>1000	24	>700

^a^PI resistance values reported previously^51^.

^b^PI resistance values determined by AlphaLISA analysis in this report from three independent experiments.

Our data illustrated multifaceted information on how these constructs responded to PI treatment. Firstly, the basal levels of precursor detection in the absence of any PI varied a little as exemplified by precursor 1319 (black lines) in Fig. 5B,C. We speculated several possible causes that are not necessarily exclusive to one and another. For example, a reduced autoprocessing activity could leave more unprocessed precursors inside cells at the steady state. Another possibility could be that the released mature PRs varied in their cytotoxicity resulting in variation in number of viable cells. Furthermore, each precursor and its autoprocessing products could have diverse activity and stability kinetics, which collectively defined the basal level signal. Because base line variation was not substantial, its impact on IC_50_ determination was considered minimal as long as there was a complete curve. Secondly, the peak height (maximum detection) varied from one to another and we also speculated involvement of multiple factors. Three precursors (NL4-3, 1319, and 6585) maintained high levels of AlphaLISA signal after reaching to the peak heights when treated with darunavir, suggesting that either the precursor amounts didn’t reach to the hook point or darunavir had minimal cytotoxicity up to 100 µM, or both combined. Under the same conditions, mutant 634 showed a decline in AlphaLISA signal when darunavir concentration was over 12 µM. The underlying cause of such a decline remained to be further defined although darunavir cytotoxicity was unlikely. Precursor expression level could be another variable, but this is difficult to be determined experimentally because both AlphaLISA and western blotting measure what are left from precursor autoprocessing in the cells. With varying responses of each precursor to PI treatment and not knowing whether and when precursor autoprocessing is fully suppressed, it was beyond the scope of this study to determine expression level of each precursor. Therefore, we focused our efforts on the left half of the curves for IC_50_ determination. Thirdly, a given precursor responded to different PIs differently. For example, mutants 1319 and 634 were significantly resistant to indinavir, one of the early PIs prescribed to many HIV positive patients, but remained responsive to darunavir, the latest and most potent PI. Taken together, this analysis allowed us to determine the IC_50_ of each PI on each precursor and subsequently calculated the fold change in IC_50_ compared to the NL4-3 control to represent PI resistance. Note that some precursors were not inhibited from autoprocessing even at the highest tested concentrations (*i.e*., 100 µM). Subsequently, their IC_50_s were reported as greater than a value that was estimated based on the incomplete curve.

The fold change values of three mutants (1319, 634, and 6585) to darunavir and indinavir determined by these two methods are very similar within <3-fold variations (Table 1), suggesting that the reported PI resistance of these constructs could be solely attributed to the precursor autoprocessing step. The other two mutants (14311 and 38129) illustrated much stronger resistance determined by AlphaLISA quantification than by PhenoSense® assessment. Because PhenoSense® assay reports mutation effects accumulated throughout viral replication and AlphaLISA specifically quantify precursor autoprocessing, we speculated that mutations associated with 14311 and 38129 not only rendered strong PI resistance at autoprocessing and but also affected biology of other steps. Indeed, these two mutants had only 4% and 3% replication capacity, compared to the NL4-3 reference^51^, making them appear to be more sensitive to PI suppression. Collectively, our data supported the notion that PI susceptibility at the precursor autoprocessing step is quantitatively correlated with PI resistance manifested in patients. This is consistent with a previous report showing that *in vitro* autoprocessing of precursors carrying multi-drug resistant mutations was either weakly responsive or fully unresponsive to PIs up to 150 µM, a practical limit at clinical settings; whereas the corresponding mature PRs remained sensitive to PI suppression^39^. Consequently, this AlphaLISA platform will provide a quick tool to quantify PI susceptibility of various precursor sequences, offering guidance in choosing effective anti-HIV regimen tailored to specific mutations.

PI resistance studies have extensively focused on mutations found in the PR coding region. We recently reported functional interplays between p6* and PR residues contributed to modulation of precursor autoprocessing^43^, and that some amino acid alterations outside the PR coding region also influence PI susceptibility^34,38^. We therefore asked whether the p6* peptide, *i.e*., the M1 region, plays any role in PI resistance. To address this question, the M1 region of these PI resistant precursors was replaced with the NL4-3 derived M1 sequence and the resulting constructs were compared side-by-side to the corresponding M1-PR constructs (Fig. 5D,E). We observed three kinds of outcomes. Precursors 1319, 6585, and 14311 showed no difference in PI responses carrying the M1 either from NL4-3 or from its molecular clone. Interestingly, the NL/634 chimeric precursor demonstrated right shifts (more resistant) than the 634 M1-PR precursor (Fig. 5D,E, dashed light yellow vs solid orange) with a fold change to darunavir more obvious than to indinavir treatment. The M1 region in 634 has a couple insertions plus few point mutations compared to the NL4-3 derived M1 sequence, suggesting that precursor autoprocessing activity could be modulated by different M1 sequences even in the context of the same mature PR sequence. The NL/38129 precursor displayed left shifts (more sensitive) than the 38129 precursor (dashed light purple vs solid purple) to both darunavir and indinavir treatment despite only six point mutations in the M1 region between NL4-3 and 38129. Collectively, our results suggested that precursor autoprocessing is not only influenced by mutations in the PR coding region but also by alterations in the p6* region, and that there might be certain interplays between p6* and PR, which play a regulatory role. This speculation is consistent with a recent report illustrating that precursor autoprocessing is context-dependent and its outcomes can be modulated by sequences outside the PR coding region^34^. In this regard, the AlphaLISA platform would provide an easy tool to further define PI resistance- conferring mutations under the context of various PR coding sequences and to determine potential interplays involved in regulation.

## Conclusions

This report describes development, validation, and execution of a cell-based functional assay for HTS drug discovery targeting HIV precursor autoprocessing. We also illustrated precursor autoprocessing as a key contributor to PI resistance. Therefore, inhibitors selectively targeting precursor autoprocessing will compensate the current cART in combating PI resistance.

## Materials and Methods

### DNA mutagenesis

The fusion precursor encoding plasmids used in this study were engineered by the standard PCR-mediated mutagenesis and molecular cloning techniques as described previously^32–34,36^. Each construct was verified by sequencing analysis.

### Cell culture and transfection

HEK 293T cells were purchased from ATCC (Manassas, VA) and maintained in DMEM medium containing 10% fetal bovine serum, 100 units/ml of penicillin G sodium salt and 100 µg/ml of streptomycin sulfate. Transfection of HEK 293T cells by calcium phosphate in multiwell plates was described previously^32,42,43^. For AlphaLISA analysis of bulk transfected cells in 384-well plates, HEK 293T cells were seeded at ~20% confluency in either 10 cm or 15 cm dishes early in the morning to allow cell attachment and growth for ~12 hours. Transfection was then carried out late in the night. For a 10 cm dish, chloroquine was added to a final concentration of 25 µM. DNA-Calcium mixture was made by first mixing a total of 6 µg plasmid DNA in 788.4 µL H_2_O followed by an addition of 111.6 µL of 2 M CaCl_2_. Then 900 µL of 2x HBS (50 mM Hepes, 10 mM KCl, 12 mM Dextrose, 280 mM NaCl, and 1.5 mM Na_2_HPO_4,_ pH 7.04~7.05) was added slowly to the DNA-Calcium mixture with gentle vortex. The resulting mixture was then added to each dish dropwise. At 9–12 hrs post transfection, the bulk transfected cells were detached by trypsin/EDTA treatment and then pooled together in DMEM medium for seeding 30 µL in each well of a 384-well plate at ~25% calculated confluency. For example, if the transfected cells in a 10 cm dish reached at 25% confluency at the time of collection, they were then suspended in (30 µL × 384=) 11.52 ml DMEM to seed a full 384-well plate at 30 µL/well. This was based on the estimate that the growth area of a 10 cm dish is approximately equal to a 384-well plate. PIs or test compounds in 20 µL DMEM were then added to each well. The transfected cells treated with up to 1.25% DMSO alone were included as the negative controls because most PIs and small molecule compounds are dissolved in DMSO as stock solutions.

### Protein purification

Mammalian expression plasmids encoding for GST-flag, GST-p6 or GST-flag-p6* were transfected into HEK 293T cells. At ~30 h post transfection, cells were harvested and lysed with buffer containing 1x PBS, 0.5% Triton X-100 plus protease inhibitor cocktail. Cell lysate was clarified and mixed with glutathione agarose beads (Sigma Aldrich, cat# G4510) for binding. After several washes, the bound proteins were then eluted with 10 mM reduced glutathione in 50 mM Tris·HCl, pH7.4. The purified proteins were resolved with SDS PAGE followed by staining with Imperial™ protein stain (ThermoFischer, cat# 24615). The concentration of protein was determined by comparing to the titrated BSA standard protein. These proteins were used to evaluate AlphaLISA detection sensitivity.

The spiking protein GST-flag-p6* used in HTS was purified from *E. Coli*. Plasmid pGEX-3X-p6* that express GST-flag-p6* fusion protein was transformed into *E. coli* BL21 cells. About 1L cultured cells were collected and resuspended in 1x PBS with 1% Triton X-100 and 1 mM PMSF, and then lysed with a Microfluidizer Processor (Microfluidics, Model M110L). The clarified cell lysates were then loaded onto a glutathione agarose column and the bound protein was eluted with 10 mM reduced glutathione in 50 mM Tris·HCl, pH 7.4. The eluted GST-flag-p6* was further purified through gel filtration twice with column HiLoad Superdex 200 (GE Healthcare Life Science) using buffer 50 mM Tris·HCl, pH 7.4, 150 mM NaCl, 2 mM DTT. The peak eluents were pooled, concentrated, and kept in aliquots at −80 °C.

### SDS-PAGE and Western blotting

To compare AlphaLISA with western blotting detection, the bulk transfected cells were seeded into either 384-well (for AlphaLISA analysis) or 24-well plates (for western blotting analysis) at 25% confluency in parallel and treated with or without PIs as indicated. At the end of a ~24 hrs incubation, the cells in 384-well plates were analyzed by AlphaLISA; the cells in 24-well plates were gently rinsed with 1x PBS once, and lysed *in situ* by adding 40 µl lysis buffer A (Tris-HCl, pH 8.0, 150 mM NaCl, 1% sodium deoxycholate, and 1% Triton X-100) with protease inhibitor cocktail. The cell lysates were collected and subjected to a brief centrifugation (10,000 × g for 2 min) to remove host chromosomes. The resulting post-nuclear supernatants were directly analyzed by western blot or stored at −20 °C. Approximately equal volumes of the post-nuclear lysates were resolved by SDS-PAGE followed by protein transfer to a PVDF membrane. Primary antibodies used in this study include rabbit anti-GST (Sigma, cat# G7781), mouse anti-flag (Sigma, cat# F1804), mouse anti-GAPDH (Millipore, cat# MAB374). Secondary antibodies included IR700 fluorescence labeled goat anti-rabbit (Rockland, cat# 611-130-122) and IR800 goat anti-mouse (Rockland, cat# 610-132-121). The blots were visualized with an Odyssey infrared dual laser scanning unit (LI-COR Biotechnology, Lincoln, Nebraska). To reduce background noise in some blots, the primary antibody was absorbed against the cell lysates made from untransfected cells that were resolved by SDS-PAGE and transferred onto a PVDF membrane. Intensity of each band was determined with Image Studio software (LI-COR Bioscience) and was then divided by the GAPDH signal of the same lane. The resulting normalized values in arbitrary unit were used to represent the protein amounts of fusion precursor, sGST -M1 and PR-Flag for comparative quantification analysis (Fig. 2).

### AlphaLISA analysis

After a ~24 hrs incubation, the culture medium was removed by a gentle aspiration and the remaining cells were lysed *in situ* by 20 µL assay solution containing 1x AlphaLISA Immunoassay buffer (PerkinElmer AL000F), protease inhibitor cocktail, 15 µg/ml anti-flag acceptor beads (PerkinElmer AL112R) and 11.25 µg/ml Glutathione donor beads (PerkinElmer 6765301). As a positive control, the purified protein GST-Flag-p6* made in 1x AlphaLISA Immunoassay buffer at 5-fold serial dilutions (8 points total) was spiked into wells only treated with DMSO. Following at least another 2 h incubation at 37 °C, each plate was read for fluorescence signal first, when needed, followed by AlphaLISA signal detection by either an EnSpire or an EnVision 2104 Microplate Reader (PerkinElmer).

### Compound Libraries

APExBio Protease Inhibitor Library (cat# L1035, APExBio) has a collection of 130 protease inhibitors dissolved in DMSO at 10 mM (100 µL/well in one and a half 96-well plates). The detailed information is available online (https://www.apexbt.com/downloader/panellist/L1035-DiscoveryProbe-Protease-Inhibitor-Library.xlsx).

The high-throughput screening and chemical biology core facility at University of Colorado Anschutz Medical Campus provided the following libraries. NIH Clinical collections 1 and 2 have 450 and 281 compounds, respectively. These compounds have well-documented clinical histories and known safety profiles. The Spectrum Collection contains 2320 compounds including approved drugs (50%), diverse natural products (30%), and other bioactive compounds (20%). The Life Diversity set (20,000 compounds) is selected from Life Chemical’s collection of over 750,000 compounds, with the application of chemical filters that exclude known toxicophores, likely assay interferers, undesirable functional groups, frequent-hitters, Michael acceptors, and other filters.

### High Throughput Screens

The APExBio protease inhibitor library was screened at 10 µM final concentration with 0.1% DMSO or 50 µM with 0.5% DMSO at duplicates. The NIH Clinical collections I and II, and the Spectrum Collection were screened at 12.5 µM with 1.25% DMSO. The elevated levels of DMSO was due to the use of daughter plates having the compounds made at 1 mM stock solution in 100% DMSO. The Life Diversity set was screened at 50 µM with 0.5% DMSO.

Each 384-well plate (Cat# 82051-278, Greiner Bio-one) could test up to 320 compounds in columns 3 through 21. We used the wells in columns 1 and 2 as negative controls by supplying DMSO to the final concentration matching to each screen library. For example, the compounds in APExBio protease inhibitor library were at 10 mM in 100% DMSO and these compounds were diluted 1000x to reach 10 µM testing concentration. Consequently, we supplied DMSO to 0.1% final concentration in DMEM culture medium in columns 1 and 2 as negative controls. In parallel, we used the wells in columns 23 and 24 as positive controls by adding PIs to the indicated concentration in the presence of DMSO that also matched to the negative controls. The screen compounds were normally diluted in the culture medium to give rise a concentration that was 2.5-fold of the targeted screen concentration. Then 20 uL of the diluted compound was added to the 384-well plate that already had 30 uL bulk transfected cells. These 384-well plates were then incubated in a CO_2_ incubator at 37 °C for ~24 hrs prior to AlphaLISA analysis.

## Electronic supplementary material

Supplementary Information

## Data Availability

The datasets generated during and/or analyzed during the current study are available from the corresponding author on reasonable request.
