# Supplementary material for: Targeting HIV-1 Protease Autoprocessing for High-throughput Drug Discovery and Drug Resistance Assessment
Source: Sci Rep. 2019 Jan 22;9:301. doi: 10.1038/s41598-018-36730-4 (PMC6343032; doi:10.1038/s41598-018-36730-4)
Supplement: Supplementary file 1 — Supplementary Information [file 41598_2018_36730_MOESM1_ESM.pdf]

Supplementary Information

Targeting HIV-1 Protease Autoprocessing for High-throughput Drug Discovery and Drug Resistance Assessment

Liangqun Huang, Linfeng Li, ChihFeng Tien Daniel V LaBabera, Chaoping Chen

Sup Fig S1

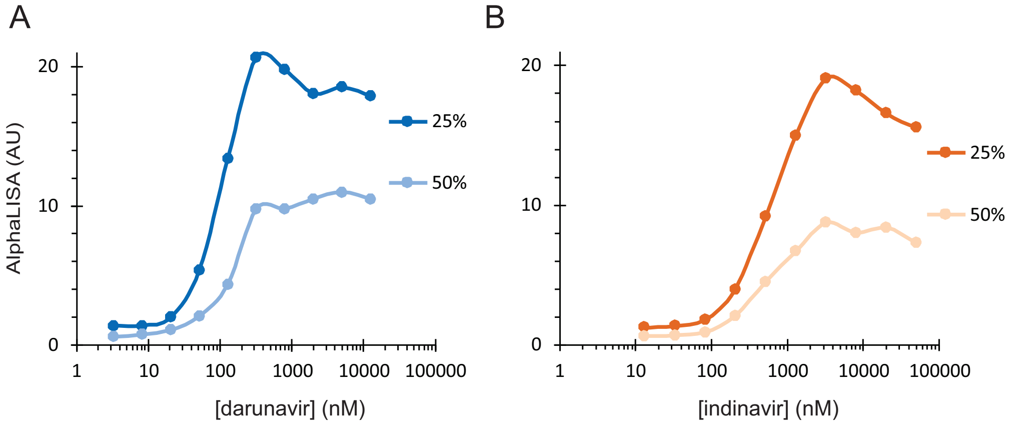

Huang et al

Sup Figure S1. AlphaLISA detection vs plating density. The bulk transfected cells were seeded in 384-well plates at the indicated confluency and treated with darunavir (A) or indinavir (B) at duplicates for about 24 hrs. AlphaLISA signal (in arbitrary unit) was then calculated and the averages were plotted as a function of PI concentration.

Sup Fig S2

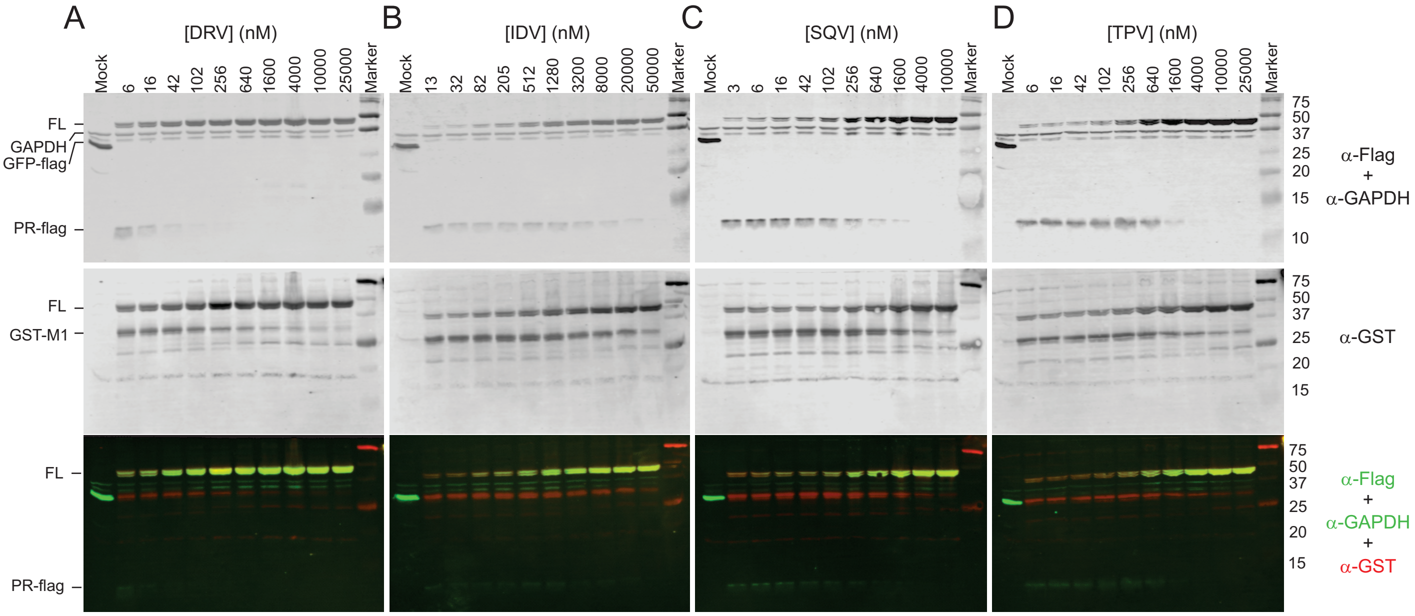

Huang et al

Sup Figure S2. Quantification of precursor autoprocessing by western blotting. The transfected cells were treated with the indicated PIs and the post-nuclear cell lysates were analyzed by SDS-PAGE and western blotting. The images were from four gels analyzed in parallel at the same time. The upper panel was visualized with mouse anti-Flag and anti-GAPDH followed by IR800 anti-mouse probing; the middle panel was visualized with rabbit anti-GST followed by IR700 anti-rabbit probing. Duel detection of IR700 and IR800 channels with a LI-COR scanning unit allowed simultaneous examination of multiple bands in the same gel. The bottom panel showed bands from both channels (red for IR700 and green for IR800) merged together. Band intensity normalized to GAPDH signal of the same lane was determined to represent protein amount for quantitative analysis.

Sup Fig S3

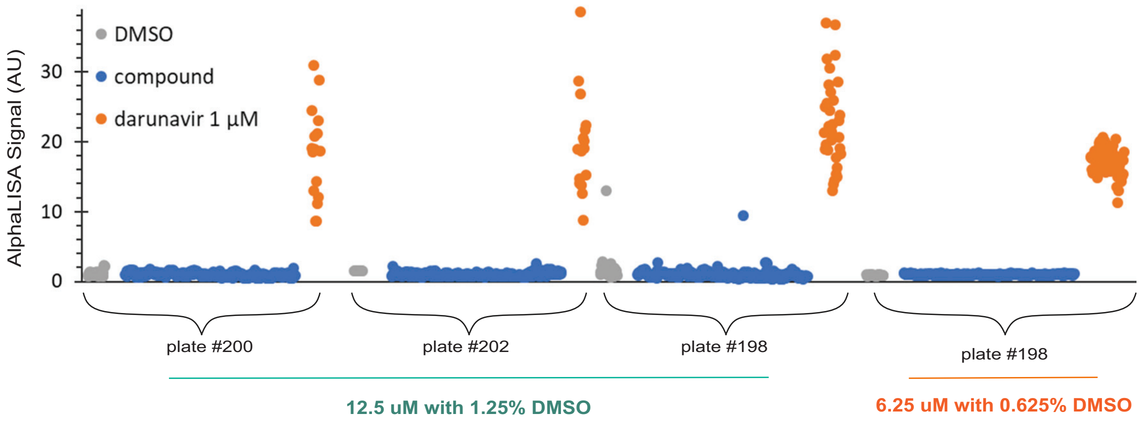

Huang et al

Sup Figure S3. DMSO effects on AlphaLISA performance. Bulk transfected cells were seeded into 384-well plates and treated as indicated. Orange dots are samples treated with 1 μM darunavir serving as the positive controls; blue dots are the test compounds; grey dots are negative controls treated with DMSO at the indicated concentrations.
